# Editor’s note: Climate change and health in aging populations

**DOI:** 10.1007/s40520-024-02832-5

**Published:** 2024-08-09

**Authors:** Nicola Veronese

**Affiliations:** Geriatric Unit, Department of Internal Medicine and Geriatrics, University of Palermo, Via del Vespro, 141, Palermo, 90127 Italy

With this editorial, I would like to discuss a paper recently published in Aging Clinical Experimental Research. As we know, dementia, a debilitating condition characterized by progressive cognitive decline, is a major public health issue, imposing significant social and economic burdens globally. With an estimated 15.07 million cases of dementia among older adults in China alone, projected to rise to 48.98 million by 2050, the urgency for effective prevention strategies is evident. The recent study conducted by Tao Zhang and colleagues sheds new light on an underexplored environmental risk factor, i.e., fine particulate matter (PM2.5) and its chemical constituents [1].

Briefly, the study’s comprehensive approach, involving 7,804 participants aged 60 and above from Zhejiang province, China, reveals critical insights into how specific components of PM2.5—namely sulfate, nitrate, ammonium, and organic matter—are linked to cognitive impairment. The findings underscore the importance of considering environmental factors, alongside genetic predispositions, in the fight against dementia.

We know that PM2.5, particulate matter with a diameter of less than 2.5 micrometers, is a pervasive air pollutant known for its harmful effects on respiratory and cardiovascular health [2]. However, its impact on cognitive health has only recently begun to garner attention. In my opinion, Zhang et al.‘s study is pivotal as it identifies specific components of PM2.5 that contribute to cognitive decline, thus offering a more detailed understanding of the pollution-cognition link. Briefly, their results indicated that long-term exposure to higher concentrations of sulfate, nitrate, ammonium, and organic matter in PM2.5 significantly increased the risk of developing cognitive impairment. Interestingly, black carbon, another component of PM2.5, did not show a significant association, suggesting that not all components of PM2.5 equally contribute to cognitive decline.

While the study establishes a clear epidemiological association, the mechanisms by which PM2.5 components affect cognitive function remain to be fully elucidated. It is hypothesized that these fine particles, due to their small size, can penetrate deep into the lungs and enter the bloodstream, potentially reaching the brain and causing neuroinflammation [1] Sulfate, nitrate, and ammonium, commonly found in industrial emissions and vehicular exhaust, may exacerbate oxidative stress and inflammation in the brain, thereby accelerating cognitive decline. Moreover, organic matter in PM2.5, which includes a variety of carbon-based compounds, may also play a role through similar pathways. The chronic exposure to these pollutants could lead to cumulative damage over time, manifesting as cognitive impairment in older adults. Understanding these mechanisms is crucial for developing targeted interventions to mitigate the impact of air pollution on brain health.

Furthermore, the findings from Zhang et al.‘s study have profound implications for public health and policy. First and foremost, they highlight the need for stringent air quality regulations to reduce the concentrations of harmful PM2.5 components. Governments and regulatory bodies must prioritize measures to curb emissions from industrial and vehicular sources, thus protecting vulnerable populations, particularly older people, from the insidious effects of air pollution. Moreover, public health initiatives should incorporate environmental risk factors into dementia prevention strategies. Awareness campaigns can educate communities about the risks associated with PM2.5 exposure and promote actions to minimize personal exposure, such as using air purifiers, avoiding outdoor activities during high pollution periods, and advocating for cleaner energy sources.

Due to the importance of this work, I am extremely happy that it broke the ice in our recent article collection entitled “Climate Change and Health in Aging Populations”. The main aim of this article collection is to publish novel works about a hot topic in medicine, i.e., climate change. Climate change may, in fact, affect not only respiratory and cardiovascular conditions as largely reported in the literature, but also geriatric syndromes, such as dementia. Therefore, the Guest Editors and I are extremely happy to receive pertinent works about this “hot” topic.
